# A cardiac hydatid cyst underlying pulmonary embolism: a case report

**DOI:** 10.4314/pamj.v8i1.71061

**Published:** 2011-02-20

**Authors:** Abid Leila, Lobna Laroussi, Med Abdennadher, Sameh Msaad, Imed Frikha, Samir Kammoun

**Affiliations:** 1 Cardiology Department, Hedi Chaker University Hospital, Sfax, Tunisia; 2 Cardiosurgery Department, Habib Bourguiba University Hospital, Sfax, Tunisia

**Keywords:** Echinococcosis, Hydatidosis, Pulmonary embolism, cardiac, hydatid cyst

## Abstract

Hydatid cysts located in the interatrial septum are especially rare but when they occur, they might cause intracavity rupture. We report on a patient with acute pulmonary embolism caused by an isolated, ruptured hydatid cyst on the right side of the interatrial septum. A 16-year-old-boy with an uneventful history was hospitalized for exercise-induced dyspnea and blood expectorations. Multiple and bilateral opacities were visualized on standard chest x-ray. Signs of right-sided hypertrophy were seen on ECG. Imaging findings led to the diagnosis of pulmonary embolism complicating cardiac hydatid cysts. An operation was performed through median sternotomy to remove the cardiac cyst. The pleural cavity was entered through the fifth intercostal space to withdraw lung hydatid cysts. Operative recovery was uneventful and the patient resumed his normal activities 19 months later. Prompt diagnosis and an appropriate surgical treatment prevented a potentially fatal outcome.

## Introduction

Cardiac hydatidosis, a potentially lethal condition, is uncommon (0.5-2% of cases) compared with the high incidence of hepatic and pulmonary involvement accounting for more than 60% and 25% of cases respectively.

The right ventricle localization is exceptional and may be revealed by cyst rupture. Hydatid pulmonary embolism generally occurs after an intracardiac rupture of the right ventricle hydatid cyst. We report our clinical and surgical therapeutic approach in a case of a patient who has developed acute hydatic pulmonary embolism due to the rupture of a right atrial hydatic cyst.

## Case report

**Figure 1:**
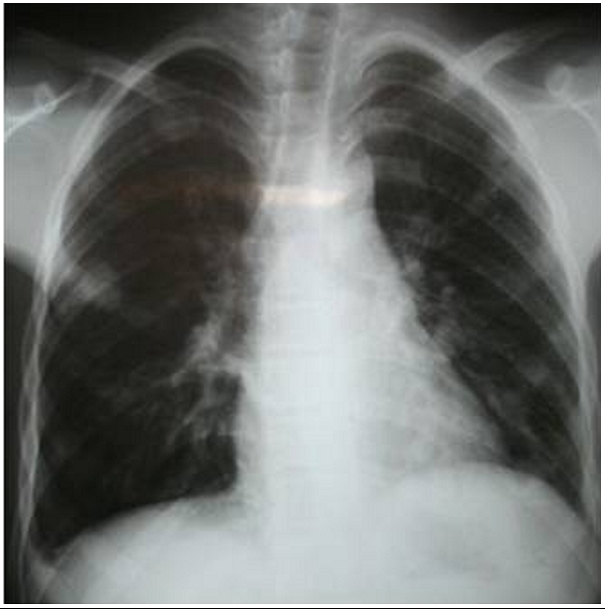
Chest X-ray showing regular masses scattered throughout both lung fields in a 16 years old Tunisian with cardiac hydatid cyst

**Figure 2:**
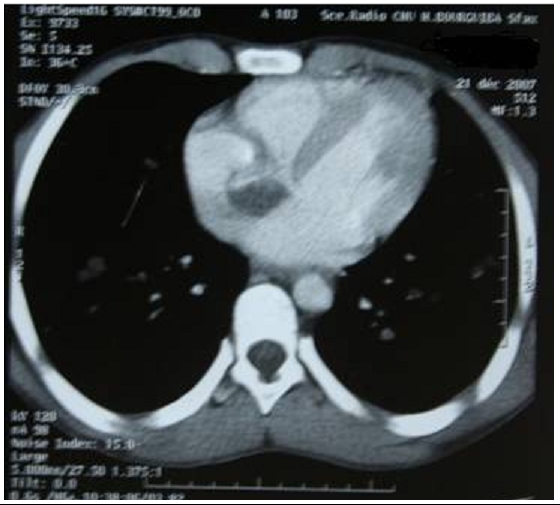
Angioscan of the chest showing multiple segmental defects with intra right atrial mass in a 16 years old Tunisian with cardiac hydatid cyst

**Figure 3:**
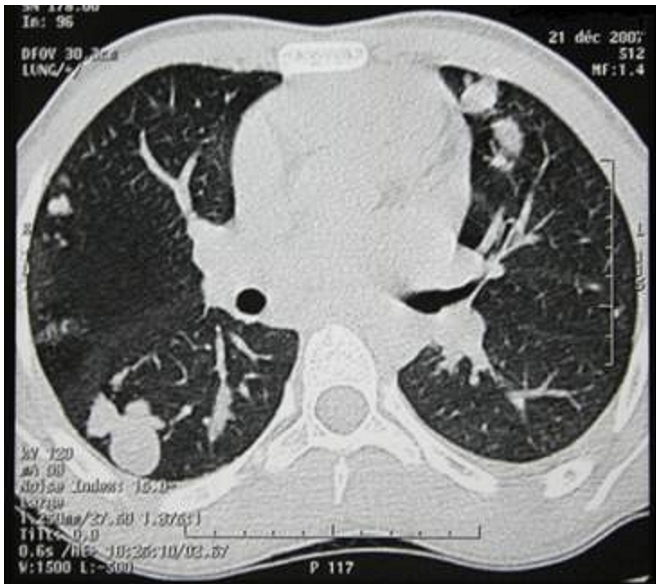
Angioscan of the chest showing multiple pulmonary hydatid ocalizations in a 16 years old Tunisian with cardiac hydatid cyst

A 16-year-old boy was admitted to our department with a history of increasing shortness of breath, chest pain and hemoptysis which had started approximately 7 days prior to admission. On admission, the patient was dyspneic and mildly cyanotic. On examination, the respiration rate was 40 breaths/ min; crepitate and bronchial rales were heard in the two lung fields. The blood pressure was 100/70 mm Hg. The pulse rate was 110 beats/ min. The neck veins were distended. The Liver and the spleen were palpable but no tender. There was no edema. The electrocardiogram revealed sinus tachycardia, incomplete right bundle block, right axis deviation, and right ventricular hypertrophy. The Chest radiography disclosed regular masses scattered throughout both lung fields (Figure 1). Laboratory results were within normal limits except for a mild eosinophilia. The titer of antiechinococcal antibodies was positive. Angioscan of the chest showed distended distal branches of the right and left pulmonary artery due to partial occlusion by cystic lesions and multiple segmental defects with multiple pulmonary hydatid localizations (Figure 2 and 3). An intra right atrial mass was also detected. Spiral CT scan of the abdomen showed normal liver, spleen, pancreas and kidneys.

**Figure 4:**
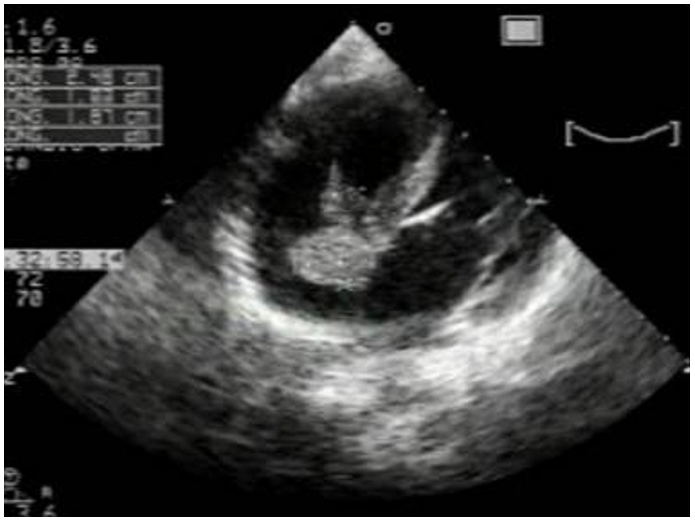
Two-dimensional echocardiography, in the apical four chamber view showing a large interatial septum cystic mass in a 16 years old Tunisian with cardiac hydatid cyst

A two-dimensional echocardiography in the apical four chamber view showed a large cystic mass measuring 19 x 22 mm, with a large implantation basis adhesive to the right side of the inter atrial septum with an extension to the right ventricle (Figure 4). No cysts in pericardial cavity were revealed. The pulmonary artery trunk, left and right branches were mildly distended without any proximal cysts.

The clinical history, the imaging findings and the prevalence of hydatid cysts in our country led to the diagnosis of a pulmonary embolism complicating cardiac hydatid cysts. No MR angiography was performed.

The patient was operated on an emergency basis. The operation was performed through a median sternotomy incision under extracorporeal circulation. The pericardium was carefully opened and a cardiopulmonary bypass was initiated under cold potassium cardioplegic arrest. The operative field was protected by sheets with hypertonic saline solution.

**Figure 5:**
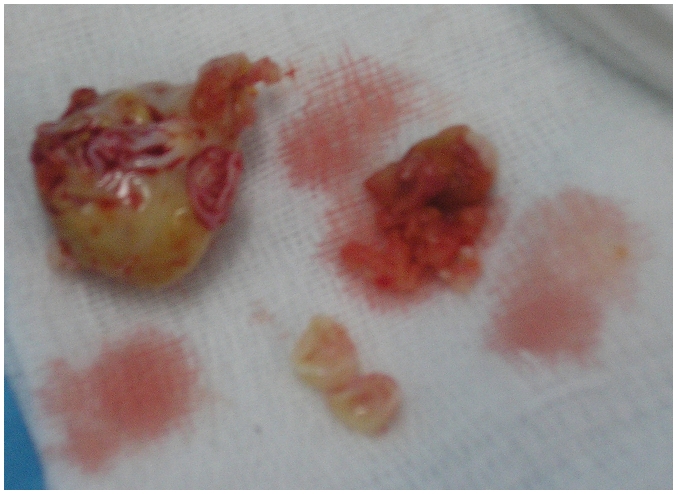
Right atriotomy showing the cyst exposed through the incision  mass in a 16 years old Tunisian with cardiac hydatid cyst

**Figure 6:**
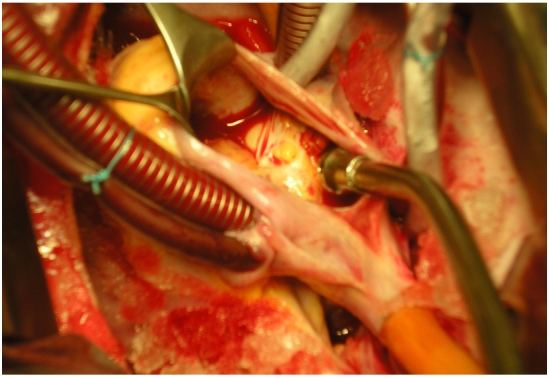
Multi vesicular atrial cystic (2 cm in dameter) mass in a 16 years old Tunisian with cardiac hydatid cyst (surgical view)

Oblique right atriotomy was performed; the cyst (2 cm in diameter) was exposed through the incision. The resection of the totality of the atrial cystic formation was performed; the content of the mass was multi vesicular (Figure 5 and 6). The interatrial septum, judged thin and delicate was strengthened by sutures. The interventricular septum explored was normal. The atriotomytomy was closed. The second step was the exploration of the pleural cavity through the fifth intercostal space and peripheral pulmonary cysts were removed. The residual cavities were closed. The postoperative course was uneventful.

The patient was discharged after 8 days and treated with Albendazole. 18 months later, he was doing well. We noticed a spectacular regression of the distal pulmonary artery cysts. The patient has a check-up every 6 months by physical examination, chest roentgenogram, echocardiography and anti-echinococcal antibodies test. There has been, until recently, no evidence of thoracic hydatidosis recurrence and no signs of chronic pulmonary hypertension.

## Discussion

Echinococcosis is an important parasitic infection that is still a common health problem in undeveloped and developing countries. Cardiac hydatid cyst disease is a rare (0, 5% to 2%) but potentially fatal pathology [1]. Cardiac hydatidosis is often primitive and unique, and it may be resulting from the rupture of a pericardium hydatid cyst [2]. In cases of heart involvement, the cyst is mostly located in the left ventricle (60%) followed by septal location [3]. Right ventricular, left atrial, right atrial and pericardial involvements have been reported. The embryo is fully grown in 1-5 years of its implantation in the heart. During this stage, the disease usually proceeds asymptomatically and can only be accidentally detected, in our case report due to a pulmonary embolism. Rupture is a lethal complication that frequently reveals a cardiac hydatid cyst. Pulmonary embolism may be due to the rupture of a hydatid cyst in the right ventricle or a venous migration of daughter vesicles to the right heart and then pulmonary arteries. Surgical and autopsy findings indicate that the embolism is caused by vesicles or daughter cysts that act purely mechanically by obstructing the blood flow and there are no blood clots or added thrombosis.

Clinical manifestations of the hydatid pulmonary embolism are not specific although hemoptisy is the most frequent sign [4]. In the absence of a medical history of a visceral hydatid cyst, clinicians should use all the means necessary to reach such a diagnosis, ie, pulmonary embolism is due to hydatid cyst. The diagnostic investigation of patients with suspected hydatid pulmonary embolism should involve a two-dimensional echocardiography, a spiral CT scan and a MRI. If there is no previous history of hydatid disease its existence can be suspected by the presence of anti-echinococcal antibodies and eosinophilia in blood tests. Eosinophilia is uncommon except for cyst rupture [5].

In our case report, pulmonary embolism was confirmed by CT scan, and its hydatid nature was guided by echocardiography. Echocardiography is the investigative procedure of choice for studying cardio pericardial hydatidosis but it rarely enables direct visualization of the pulmonary embolus, which was the case of our patient. With transoesophageal echocardiography, it is possible to visualize massive emboli in the central pulmonary arteries [6]. Spiral CT scan and MRI have been used successfully in the diagnosis of hydatid cysts of the lungs and the heart. However, MRI has an advantage over spiral CT-scan in examination of the heart and the great vessels, because with images in multiple phases it gives a more complete anatomical picture [2]. Nowadays, MR-angiography in hydatid pulmonary embolism whether performed before or after surgery, yields good results [7]. Although mebendazole and albendazol have shown promise in the treatment of hydatid disease, cardiac hydatid disease should be treated surgically. The operative risks are minimal and the operation allows exploration of the cardiac cavities. The results of surgical treatment are excellent with complete recovery in the majority of cases.

Median sternotomy with cardiopulmonary bypass remains the surgical approach of choice in the treatment of the cardiac hydatic cyst and removal of echinococcal material from pulmonary arteries. The sternotomy can also allow a simultaneous treatment of both lung hydatid and cardiac cysts [8].

Treatment of the residual cavity constitutes a delicate step [9], and our policy is to use the gelatin–resorcin–formalin (GRF) glue so as to efface completely the residual cavity without deforming the ventricular cavity by an important number of sutures. Lioulias and colleagues [10] preferred the approach of left thoracotomy, because there were no cysts within the cardiac chambers or the main pulmonary trunk and the main site of embolism was in the left pulmonary artery [11].

## Conclusion

A high index of suspicion is required to diagnose hydatid pulmonary embolization. Early diagnosis, particularly in the acute form of pulmonary embolization complicating cardiac location, is of utmost importance because surgical intervention is the only treatment that could is life saving.

## Competing interests

The authors declared no competing interests

## Authors’ contribution 

All the authors contribute to the management of the patient and the writing up of the manuscript.
